# “Modulating Phosphoinositide Profiles as a Roadmap for Treatment in Acute Myeloid Leukemia”

**DOI:** 10.3389/fonc.2021.678824

**Published:** 2021-05-24

**Authors:** Stefano Ratti, Camilla Evangelisti, Sara Mongiorgi, Alessia De Stefano, Antonietta Fazio, Francesca Bonomini, Matilde Y. Follo, Irene Faenza, Lucia Manzoli, Bhavwanti Sheth, Magdalena C. Vidalle, Scott T. Kimber, Nullin Divecha, Lucio Cocco, Roberta Fiume

**Affiliations:** ^1^ Cellular Signalling Laboratory, Department of Biomedical Sciences (DIBINEM), University of Bologna, Bologna, Italy; ^2^ Inositide Laboratory, School of Biological Sciences, Faculty of Environmental and Life Sciences, University of Southampton, Southampton, United Kingdom

**Keywords:** phosphoinositides, PLCB1, PI3K, PIP4K, AML, epigenetic, transdifferentiation, bioinformatic

## Abstract

Polyphosphoinositides (PPIns) and their modulating enzymes are involved in regulating many important cellular functions including proliferation, differentiation or gene expression, and their deregulation is involved in human diseases such as metabolic syndromes, neurodegenerative disorders and cancer, including Acute Myeloid Leukemia (AML). Given that PPIns regulating enzymes are highly druggable targets, several studies have recently highlighted the potential of targeting them in AML. For instance many inhibitors targeting the PI3K pathway are in various stages of clinical development and more recently other novel enzymes such as PIP4K2A have been implicated as AML targets. PPIns have distinct subcellular organelle profiles, in part driven by the specific localisation of enzymes that metabolise them. In particular, in the nucleus, PPIns are regulated in response to various extracellular and intracellular pathways and interact with specific nuclear proteins to control epigenetic cell state. While AML does not normally manifest with as many mutations as other cancers, it does appear in large part to be a disease of dysregulation of epigenetic signalling and many novel therapeutics are aimed at reprogramming AML cells toward a differentiated cell state or to one that is responsive to alternative successful but limited AML therapies such as ATRA. Here, we propose that by combining bioinformatic analysis with inhibition of PPIns pathways, especially within the nucleus, we might discover new combination therapies aimed at reprogramming transcriptional output to attenuate uncontrolled AML cell growth. Furthermore, we outline how different part of a PPIns signalling unit might be targeted to control selective outputs that might engender more specific and therefore less toxic inhibitory outcomes.

## Acute Myeloid Leukemia

Acute myeloid leukemia (AML) is a cancer of blood cells, in which myeloid progenitor cells lose their ability to differentiate while increasing their rate of proliferation giving rise to too many and/or too immature myeloid cells derivatives. These blast cells fail to differentiate into granulocytes or monocytes. Blast accumulation in the absence of proper haematopoiesis leads to impairment of the immune system and eventually to death. Although, compared to other types of cancers, AML is characterised by a low number of mutations, it is a highly heterogenous hematologic disease classified into many different subtypes, reflected in different clinical manifestations: understanding the relevance of this heterogeneity is critical to develop novel and more personalized clinical therapies (1). Today, the first line of treatment is still chemotherapy, with a 5-year survival rate of less than 30%: it is now clear that treating all patients with the same starting protocol (i.e. “one size fits all” strategy) benefits only a specific group of patients. Personalized therapies are aimed at using novel insights into patient specific genetic signatures of AML to define strategies to treat the AML (1, 2). Compared to the last 50 years, when administration of cytarabine and anthracyclines was the only standard therapy, the last 6 years has seen the introduction of many novel successful clinical trials, aimed to stratify and treat patients in subgroups tailored on patient specific genomic backgrounds (3). Although new molecular therapies are showing promising results, patients often relapse and therefore more therapeutic strategies are required. We propose that by more fully understanding how polyphosphoinositides (PPIns) profiles impact on cell behaviour, and by targeting the enzymes that modulate these lipids, a roadmap in the cell state could be generated to lead to new therapeutic strategies in AML treatments. This review will focus on how phosphoinositides participate in the regulation of cellular processes important in AML, i.e. cell growth, differentiation, apoptosis and epigenetic behaviour and how regulation of their modulating enzymes can be a beneficial additive for a more personalized AML treatment.

## Phosphoinositides

**Figure 1:**
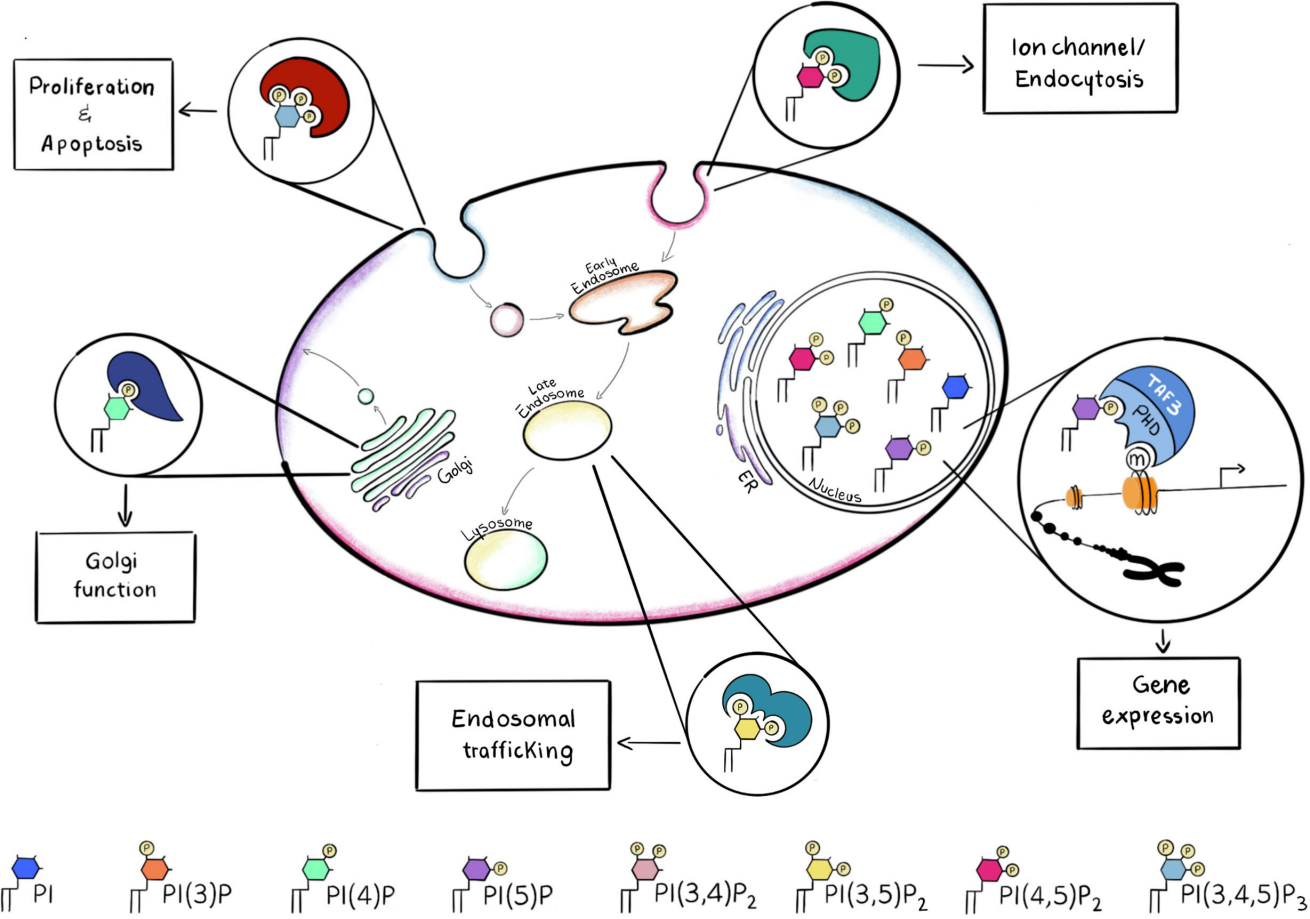
PPIns may be phosphorylated on the 3, 4 and 5 positions to generate seven possible PPIns, each with a different spatial occupancy, that is recognized by different effector proteins. Recruitment of the necessary effector protein is guaranteed by the different accumulation of PPIns in the subcellular compartments. Here are some examples of how different PPIns interact with different effector proteins to convert chemical diversity into diverse functional functions.

Phosphoinositides are a family of phosphorylated lipid molecules that directly control several essential cellular processes, such as proliferation, survival, adhesion, vesicular trafficking and transcription (4). They are derived from phosphorylation of the parent precursor molecule, phosphatidylinositol (PtdIns) and can generate seven well characterised polyphosphoinositides (PPIns) (5). The structural basis of PtdIns consists of an inositol head group linked to diacylglycerol (DAG) by a phosphodiester bond. The inositol head group of PtdIns can be reversibly phosphorylated at the 3, 4 and 5 positions of the inositol ring giving rise to 7 different molecules: phosphatidylinositol 3-phosphate (PtdIns3P), PtdIns4P, PtdIns5P, PtdIns 3,4-bisphosphate [PtdIns(3,4)P_2_], PtdIns(3,5)P_2_, PtdIns(4,5)P_2_ and PtdIns 3,4,5-trisphosphate [PtdIns(3,4,5)P_3_]. PPIns can be interconverted by the activity of kinases and phosphatases whose activations can be controlled by extracellular and intracellular inputs (6, 7). Regulation of these kinases and phosphatases at different subcellular locations can lead to organelle specific PPIns profiles which in combination with their ability to interact with specific downstream signalling proteins enables the conversion of the chemical PPIns profiles into diverse function outputs (8) (**Figure 1**).

For instance, PtdIns(4,5)P_2_ is mainly concentrated at the plasma membrane and it is involved in the regulation of integral membrane proteins such as ion channels which contain arginine lysine patches that bind to PtdIns(4,5)P_2_ and induce a change in conformation and channel activity. PtdIns(3,4,5)P_3_, which is synthesised by the phosphorylation of PtdIns(4,5)P_2_ is also predominantly localised to the plasma membrane and can initiate many different signal cascades. In contrast PtdIns(4)P is predominantly found in the Golgi complex (9), where it modulates Golgi structure and function, whilst PtdIns(3)P and PtdIns(3,5)P_2_ are found predominantly within early and late endosomes or lysosomes (10) (**Figure 1**). PtdIns(3,4)P_2_ is another key molecule, when localized at the plasma membrane it can assist cytoskeletal rearrangements important for clathrin mediated endocytosis, macropinocytosis and lysosomal catabolism, cell migration and, in cancer cells, invasion (11). Moreover PtdIns(3,4)P_2_ can mediate glucose uptake and insulin signalling and become a key second messenger (11).

After binding PPIn, proteins can change their localisation, conformation, interaction partners and also activity so that these interactions control various cellular processes (8): here we will describe some of them, relevant for AML.

## Phosphoinositide 3-Kinases and AML

**Figure 2:**
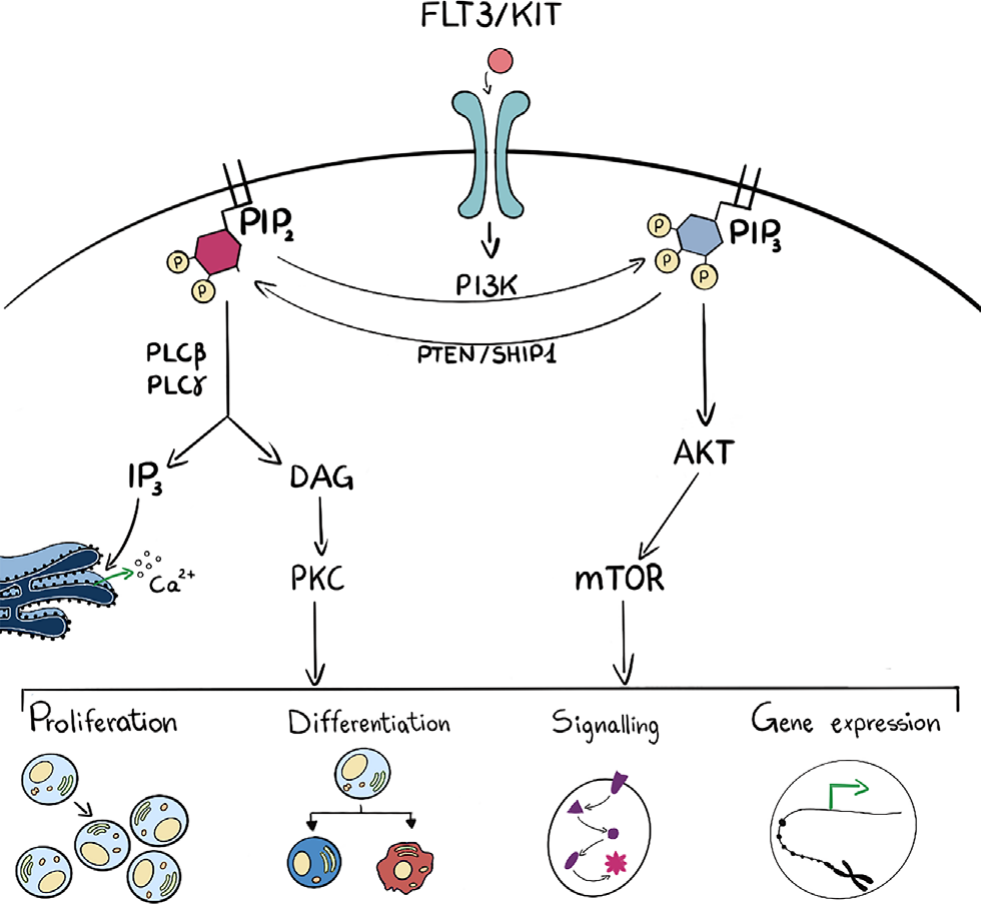
Schematic representation (on the left side) of PLC activation which hydrolyses PIP_2_ into DAG and IP_3_. These last two are intermediate second messengers that can regulate many cell functions such as proliferation, differentiation, signal transduction and gene expression. On the right side, ligand binding to membrane receptor drives PI3K activation and consequent phosphorylation of PIP_2_ into PIP_3_ and subsequent activation of the AKT/mTOR pathway. These activated players can as well regulate many cell functions such as proliferation, differentiation, signal transduction and gene expression. PIP_3_ can be dephosphorylated to PIP_2_ by phosphatases such as PTEN and SHIP1. Many intermediate players are omitted for figure clarity.

Phosphoinositide 3-kinases are lipid kinases that phosphorylate one or more inositol phospholipids on the 3-position of the inositol ring (**Figure 2**). There are eight PI3Ks in mammalian cells that are sub grouped into three unique classes based on structural and enzyme-kinetic differences; four Class I isoforms (PI3K -α, -β, -γ, -δ), three Class II isoforms (PI3K-C2α,-C2β and -C2γ) and a single Class III isoform, known as vacuolar protein sorting 34 (Vps34) (12). In particular, PI3Kγ and PI3Kδ isoforms are abundant in hematopoietic cells, such as leukocytes (13, 14), whilst PI3Kα and PI3Kβ are mainly ubiquitously expressed. Class I PI3K can phosphorylate PtdIns(4,5)P_2_ into PtdIns(3,4,5)P_3_ and increased levels of PtdIns(3,4,5)P_3_ are sensed by specific Plekstrin Homology (PH) domain containing proteins such as the serine threonine kinase AKT/PKB. PtdIns(3,4,5)P_3_ can be degraded by a number of phosphatases such as PTEN, SHIP1 or INPP5D which are essential to maintain long term hematopoietic stem cells (15). Interaction with PtdIns(3,4,5)P_3_ leads to activation of kinases and phosphatase that exert a wide spectrum of effects on downstream pathways which include cell proliferation, differentiation, apoptosis and metabolism (16). Class II isoforms have distinctive, non overlapping functions that regulate cell migration, proliferation and survival (17). PI3K-C2α is also involved in PtdIns(3,4)P_2_ -mediated vesicular trafficking, membrane remodelling important for platelet formation and can be a scaffold protein important during mitosis. PI3K-C2β is an activator of Ca^2+^ flux and regulates many signalling pathways, whilst PI3K-C2γ is mainly involved in vesicular trafficking and in glucose homeostasis (17).

The ubiquitous utilization of PI3K signalling by diverse receptor families together with the identification of mutations in multiple components of the PI3K signalling pathway in various cancers, has led to the development of compounds targeting this pathway. Many of these are under clinical investigation for cancer treatment showing varied levels of success (18).

In AML, the PI3K pathway dysregulation is a frequent event and correlates with poor prognosis (19). Constitutive activation of PI3K signalling is associated with hematologic malignancy and is probably triggered by abnormal activations of KIT, FLT3 and RAS, which are frequently muted in AML (20, 21).

Several preclinical trials using PI3K inhibitors have progressed to clinical trials but even though PI3K-related inhibition may target AML cells, including Leukemic Stem Cells (LSCs), they have met with a very limited success as monotherapies, probably as a consequence of compensatory activation of other survival pathways (22). For this reason, novel combinations and alternative pathway inhibitions may result in more efficacious and tolerable pharmacological regimens for AML treatment.

Firstly, PI3K/mTORC1/2 inhibition reached higher apoptosis rates than single inhibition or combined AKT/mTORC1 inhibition. Primary patient samples and cell lines carrying MLL rearrangements show higher sensitivity to PI3K/mTOR inhibition. In a larger cohort of MLL-AF9+ AML patients, a high incidence of additional mutations in genes involved in growth factor signalling pathways was identified, which could explain their preferential sensitivity (23). In THP-1 cells and patient-derived xenografted (PDX) cells, a combination treatment of the dual PI3K/mTORC1/2 inhibitor BEZ-235 with a MEK inhibitor showed highly synergistic effects on apoptosis. Using the MLL-AF9+ xenograft mouse model, Sandhöfer et al. highlighted the efficacy of PI3K/mTORC1/2 inhibition *in vivo*. Altogether, these data show a possible benefit of PI3K/AKT/mTOR inhibition as a therapeutic approach for MLL-rearranged leukaemia.

In the battle against AML, the effect of PI3K/AKT/mTOR inhibition can be augmented by BCL-2, which is an anti-apoptotic protein often overexpressed in several blood disorders, including AML. Increased expression of BCL-2 enhances survival by blocking apoptosis and is associated with increased chemoresistance and poor patient outcome (24).

The selective BCL-2 inhibitor Venetoclax has shown strong cytotoxic effects combined with a safe patient profile in AML (25) and Venetoclax has recently been approved by the FDA for CLL patients with the 17p deletion (26). Furthermore, it entered phase II clinical trials as a monotherapy in patients with refractory and relapsed AML (27).

Complete responses to BCL-2 are only observed in approximately 20% of patients suggesting that monotherapy does not reach durable responses. Thus, Venetoclax combined with inhibitors of survival pathways or classical chemotherapeutic drugs have been assessed for the treatment of AML, to increase the cytotoxic effects. Among these, the combined treatment of BCL-2 and PI3K inhibition enhanced leukemia cell death in AML cell lines, patient-derived blasts and xenograft models (28). The anti-leukemic effects of this drug combination can be further increased by ERK inhibition (29) although how toxicity profiles are affected is yet to be assessed. Downregulation of Mcl-1, a BCL-2 related antiapoptotic protein with PI3K/mTOR inhibitors may underlie the potentiation of the effect of Venetoclax, in leukemia cells (30).

## Phospholipase and AML

Phospholipases C (PLCs) hydrolyse PtdIns(4,5)P_2_ into DAG and Ins(1,4,5)P_3_: DAG can be bound by many proteins, i.e. protein kinase C (PKC) that transduce changes in DAG levels into phosphorylation and regulation of downstream targets, whilst Ins(1,4,5)P_3_ can be bound by its cognate receptor on the endoplasmic reticulum and regulate Ca^2+^ efflux (**Figure 2**). Phospholipases are localized mainly at the plasma membrane but also in cell organelles: for example PLCβ1 is present in the nucleus where it is involved in transcriptional regulation (31–35).

Interestingly, PLCβ1 is involved in haematological malignancies by regulating haematopoiesis, especially in Myelodysplastic Syndromes (MDS), where it acts on both erythropoiesis and myelopoiesis (36–38), with possible implications in transformation into AML. PLCβ1 modulation is clinically relevant in leukemogenesis of MDS, as its mono-allelic deletion is associated with increased risk of AML progression and its expression is inversely correlated with AKT/ mTOR activation in higher-risk MDS (39–42). Moreover, specific mutations in inositide regulating enzymes including PLCγ2, AKT3 and PIK3CD were associated with Azacytidine and Lenalidomide therapy failure in MDS leading to a higher risk of AML progression (43). In addition to PLCβ1, also PLCβ3 plays a crucial role in haematopoiesis, since PLCβ3-deficient mice develop myeloproliferative disease, lymphoma, and other tumors (44): these mutant mice have increased numbers of hematopoietic stem cells with increased proliferative, survival and myeloid-differentiative abilities. Particularly, PLCβ3 exerts this function, not by its lipase activity, but by being a scaffold protein that hold together SHIP1 and the transcription factor Stat5, that in turn regulates the above processes.

Recent studies have observed also that survival of Leukemic Stem Cells from the bone marrow of AML patients is dependent on ORP4L, a protein that acts to scaffold PLCβ3 into a complex at the plasma membrane. ORP4L is able to extract PtdIns(4,5)P_2_ from the plasma membrane and presents it as a substrate to PLCβ3 for hydrolysis, mediating Ins(1,4,5)P_3_-induced endoplasmic reticulum Ca^2+^ release (45, 46). Importantly, genetic or pharmacological inhibition of ORP4L leads to LSCs death in AML and to defective bioenergetics, autophagic death and abrogation of T-ALL engraftment *in vivo* (47).

Finally, by a peptide microarray profiling array, in a t (8, 21) AML, PLCγ1 was found hyper-expressed and PLCγ1 KD showed a decreased in AML cell growth, increase of apoptosis and a higher chemosensitivity to the chemotherapeutic drug treatments upon hypoxic stress (48).

## Phosphatidylinositol-5-Phosphate 4-Kinase and AML

In a recent study a sh-RNA library targeting modulators of PPIns was used to identify novel targets essential in AML proliferation in at least three different AML cell lines. From the screen common modulators emerged as essential for proliferation and included three PPIns phosphatases (INPP5J, INPP5B, SYNJ), subunits of the PI3K pathway (PI3K-C2α, PI3K-R3, PI3K-R6), PLCβ2 and PIP4K2A (49). PIP4K2A is a kinase that phosphorylate PIns or Pins5P on the 4-position of the inositol ring, thus regulating the levels of both PtdIns(4,5)P_2_ and PtdIns5P. Silencing PIP4K2A attenuated growth of primary human AML cells, while sparing healthy Hematopoietic Stem Cells HSCs (49). In AML cells, PIP4K2A regulates cell cycle progression and apoptosis dependent on the activation of mTOR and represents a novel potentially druggable target for the treatment of AML. The pro-leukemic role played by PIP4K2A was also demonstrated in paediatric acute lymphoblastic leukaemia, where its expression correlated with chemoresistance (50). Moreover, susceptibility for development of acute lymphoblastic lymphoma has been associated with SNPs in both intronic and exonic regions of the PIP4K2A gene (51, 52).

The revelation that many different PPIns modulators are essential for AML cell growth suggest that understanding their individual mechanism of action might lead to the development of patient specific therapies that could be generated through combinations of molecules that deregulate these pathways. Importantly the enzymes that modulate PPIns are highly druggable and many inhibitors are already available. Given that many of these enzymes are also found in the nucleus where they regulate a specific pool of PPIns, which impact on transcriptional output we next describe their potential roles as epigenetic regulators in AML.

## The Potential for Targeting Nuclear Phosphoinositides as Epigenetic Regulators in AML

PPIns and in particular PtdIns, PtdIns4P, PtdIns5P, PtdIns(4,5)P_2_ and PtdIns(3,4,5)P_3_ are localized in the nucleus (53), in the nuclear envelope and in the nucleoplasm. Within the nucleoplasm PtdIns(4,5)P_2_ and PtdIns4P have been localised by immunostaining to splicing speckles, nucleoli and to nuclear lipid islets (54). In the nucleus, the levels of PPIns respond to specific stimuli, such as cell stress, DNA damage, cell cycle progression or cell differentiation (55). These changes occur distinctly and independently of changes in the cytoplasmic profile of PPIns. Changes in nuclear PPIns appear to be particularly prevalent during control of differentiation or proliferation. The levels of nuclear PLCβ1 decrease during hematopoietic differentiation and increase during liver regeneration (34, 56). Surprisingly, increased nuclear PLCβ1 is required during myogenic differentiation which may be related to the initial phase of differentiation.

How exactly nuclear PPIns control differentiation is not completely clear. Nuclear PPIns can be bound by specific nuclear protein domains found in enzymes that control epigenetic signalling. For example the PHD finger domain is a nuclear receptor for PPIns, found mainly in nuclear proteins which are involved in all aspects of epigenetic signalling (57, 58). These domains also mediate interaction with modified and unmodified histone tails and act as protein dimerization domains which are likely modified by PPIns interaction. For example, the PHD fingers of ING2 (INhibitor of Growth protein 2) and TAF3 (TATA-Box Binding Protein Associated Factor 3) both act as sensors for H3K4me3 and for nuclear PPIns. ING2 regulates p53 acetylation and transcriptional output and TAF3 is a component of the basal transcription complex. In both cases, loss of PPIns interaction through mutation of the PHD finger, leads to protein loss of function, even if the interaction with H3K4me3 is unchanged. In the case of ING2 this leads to decreased acetylation of p53 and aberrant transcriptional output (57) while in the case of TAF3, which is involved in myogenic differentiation, there is a decrease in myogenic gene transcription and differentiation (59). Nuclear PPIns actually interact with a much wider variety of nuclear proteins involved in transcriptional output (60). For example, nuclear PPIns interact and allosterically regulate the Ubiquitin-like PHD and RING finger domain-containing protein 1 (UHRF1) (61). UHRF1 is a multidomain protein that regulates DNA methylation in response to changes in histone modification through its ability to interact with the DNA methylase DNMT1 and bind modified histones. The interaction between UHRF1 and PPIns occurs through a polybasic region (PBR) which are abundant in nuclear proteins. In fact, mass spectrometry revealed that PBRs were the most highly enriched domain after affinity purification of nuclear proteins on PtdIns(4,5)P_2_ beads (62). Moreover nuclear PtdIns(4,5)P_2_ also regulates the activity of the histone lysine demethylase PHF8 to control ribosomal RNA transcription (63) and the activity of chromatin remodeling complexes (64, 65). These data suggest that targeting nuclear PPIns signalling could be specifically used to control epigenetic signalling to impact on transcriptional output.

Despite having a low mutation burden, AML is highly heterogenous due to deregulation of the epigenetic landscape suggesting that epigenetic modulators could be leveraged to target and treat AML (66). On average 70% of patients have mutations in genes encoding epigenetic regulators including chromatin modifying genes or genes involved with the regulation of DNA methylation (67, 68). In Myelodysplastic syndromes (MDS), mutations are often associated with increased DNA methylation and demethylation therapies, such as azacytidine and more recently decitabine (DNA methylation inhibitors) are used to treat this disease. Interestingly, expression of nuclear PLCβ1 is increased during demethylation therapy in MDS patients and is associated with a good response to the drug (69) while monoallelic deletion of PLCβ1 is associated with poor prognosis of MDS patients (70). Other broad-based epigenetic therapies include inhibition of Histone deacetylases (HDAC). Newer more specific targets include IDH1 (isocitrate dehydrogenase 1) (71–75), DOT1, (a H3K79 methylase) and KDM1A (LSD1) a nuclear amine oxidase homolog that demethylates mono- and dimethylated Lys 4 and Lys 9 of histone H3 (76–82).

**Figure 3:**
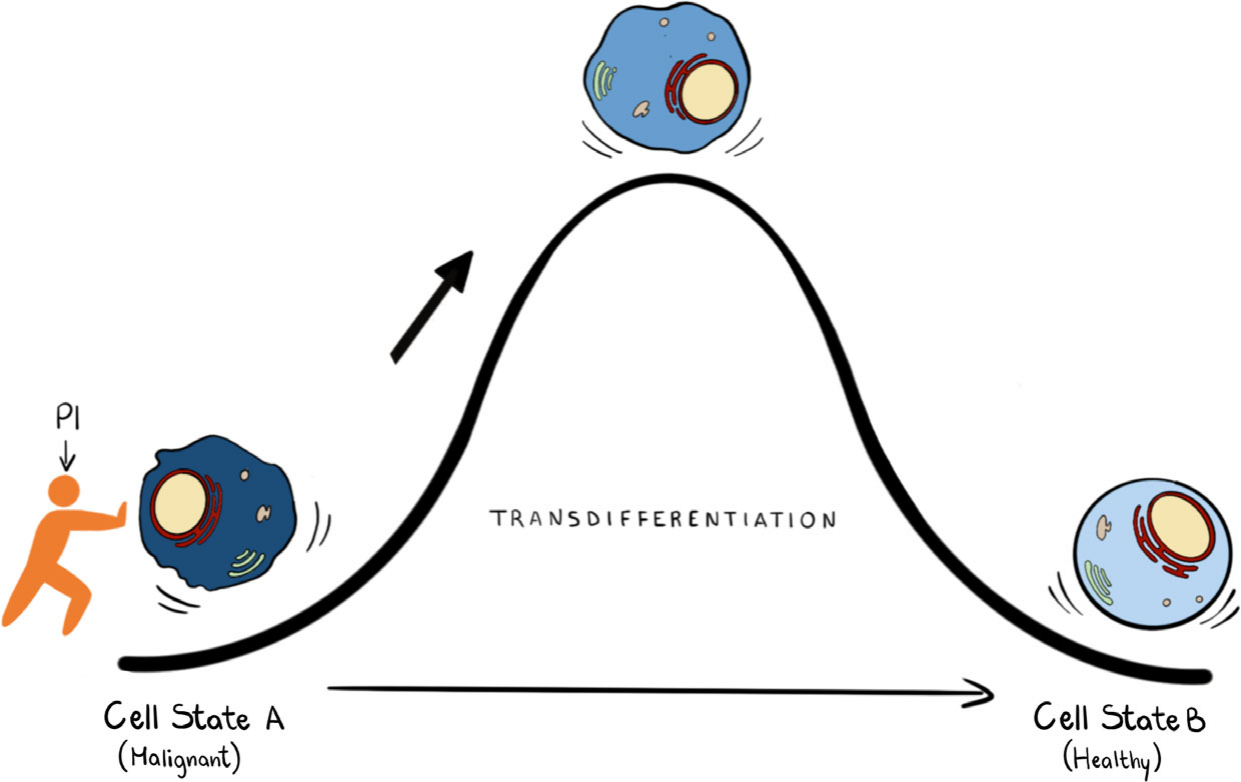
In transdifferentiation a somatic cell can pass from a somatic differentiated change to: this process can be achieved by knowing the transcription factors and the epigenetic regulators that can drive this transition. We propose that PPIns, by being able to regulate epigenetic and transcription factors, could help this transition.

Epigenetic reprogramming can also be used to unlock the potential of well-established AML therapies such as All Trans Retinoic Acid (ATRA) for its broader use as a therapeutic in AML. ATRA is highly successful in treating Acute Promyelocytic Leukaemia (APL) but has little effect in other AML subtypes. Recent studies have shown that treatment with either KDM1A or GCN5 (a histone h3K9 acetylase) inhibitors reprogram non-APL AML cells to become sensitive to ATRA induced differentiation (83, 84). Identifying novel small molecules that can induce differentiation is a difficult, lengthy and stochastic process which can be assisted by predictive computational algorithms, such as Mogrify, that combine data from RNA expression and epigenetic landscape to predict perturbations necessary to change cell state (85). Given that nuclear PPIns metabolism is intimately linked with control of epigenetic signalling and the enzymes that regulate PPIns are highly druggable it might be possible to identify combinations of inhibitors aimed at targeting PPIns metabolism to establish a particular quiescent cell state (**Figure 3**) given a deep understanding of how perturbing PPIns metabolism impact on gene expression.

Targeting epigenetic signalling enzymes, such as methylases or demethylases, may turn out to have toxicity issues and uncovering how they are controlled would allow for further fine tuning. Remarkably it appears that nuclear PPIns interaction with downstream targets regulates selective output signalling. For example, mutations that attenuate the interaction of the PHD finger of either ING2 or TAF3 with PPIns do not completely inhibit their function but rather lead to a more selective inhibition that impacts on the transcriptional output of a subset of gene targets (59, 86). PPIns interact with many different PHD and PBR containing proteins that span epigenetic writers, erasers and readers and the demonstration that therapeutically useful small molecules can be generated to block interactions between lipids and their cognate interaction domains (87) suggest that highly specific allosteric regulators that modulate the interaction of nuclear PPIns with specific proteins could be generated. These molecules are likely to selectively modulate the function of nuclear PPIns binding proteins enabling more specific targeting compared to inhibition of the PPIns modulating enzymes or of the epigenetic signalling activity itself. Combining this understanding with bioinformatic analysis as described above might enable the development of combination therapies that can subtly tune transcriptional output to drive AML tumour cells to express differentiation specific genes to attenuate proliferation.

## Conclusion and Perspectives

**Figure 4:**
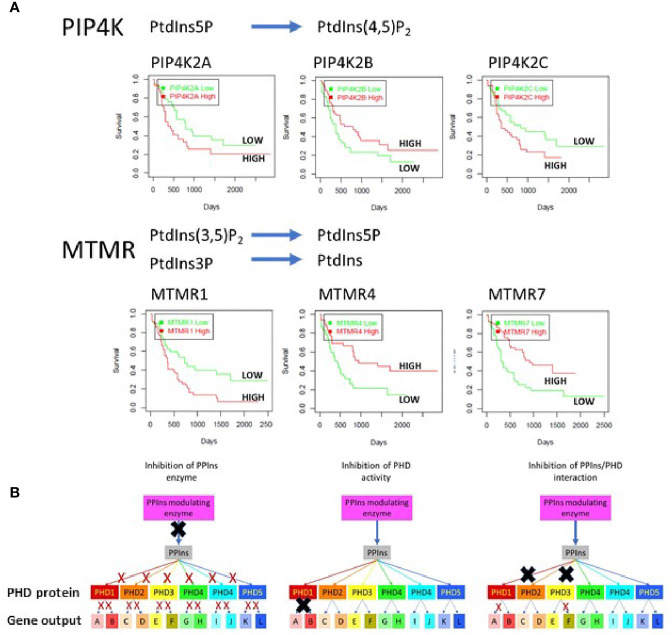
**(A)** The TGCA AML patient data base was used to assess survival outcomes with respect to low or high expression of various PPIns modulating enzymes to demonstrate complex signalling and outcomes within enzymes from the same family. As examples we shown that low expression of PIP4K2A, PIP4K2C or MTMR1 is associated with increased survival. Surprisingly however the expression of PIP4K2B, a family member with PIP4K2A and 2C shows survival outcomes that are opposite with respect to its expression. Similar data are observed MTMR4 and 7 where high expression is associated with increased survival in AML. These data suggest that the complexity in PPIns signalling could be exploited to generate modulatory pathways that could be beneficial for AML treatment. **(B)** A schematic diagram illustrating that PPIns inhibitors could act at 3 points within a PPIns signalling unit: (i) inhibition of the PPIns modulating enzyme will impair all downstream targets and associated gene expression programs (A:L) (ii) inhibition of the activity of a PHD containing protein will likely impair all the genes that the protein regulates (gene A and gene B) or (iii) allosteric inhibition of in the interaction of PPIns with a specific interacting domain will impair selected output which might be tuned to specific pathways and therefore engender less toxic outcomes. In this example inhibition of two PHD domain interactions is shown which impair expression of gene A and gene F.

PPIns regulate a vast array of cellular process impacting on nearly every aspect of biology. They are controlled by a panel of kinases, phosphatases and phospholipases which generate distinct sub-cellular PPIns profiles that impact on downstream signalling through interaction with specific target proteins. Key to the possibility of targeting PPIns in AML is that many of the enzymes that modulate PPIns are essential for growth and proliferation of AML cells but have much less impact on the growth and differentiation of normal hematopoietic stem cells. Interestingly the level of expression of many PPIns modulating enzymes also stratify AML patients in terms of overall survival. We interrogated the TCGA data base for AML patients with all PPIns modulating enzymes and found twenty-two modulators that significantly (<0.05) stratify patient overall survival. For example and in accordance with our previous studies (49), high expression levels of PIP4K2A associate with poor survival (**Figure 4A**). PIP4K2A is one of three isoforms of PIP4K that phosphorylate PtdIns5P to generate PtdIns(4,5)P_2_. Interestingly, the other two isoforms, 2B and 2C also stratify patient survival with 2C showing similar characteristics as 2A. However, PIP4K2B, which is predominantly localised in the nucleus, shows an inverse correlation with survival compared to 2A and 2C, such that low levels of 2B associate with poorer survival. This is in accord with our previous studies in breast cancer patients which revealed that low PIP4K2B is associated with poor survival (88). Similar stratification differences between PPIns modulating enzymes from the same enzymatic family are also observed in the MTMR family, which dephosphorylate PtdIns3P and PtdIns(3,5)P_2_ to generate PtdIns and PtdIns5P respectively (**Figure 4A**). While these data are difficult to interpret, they suggest the presence of exploitable complexity within the system.

Another key aspect in targeting PPIns pathways, is that the enzymes that modulate them are highly druggable and many of them have already been under intense development for the identification of small molecular weight inhibitors. For example, there are multiple inhibitors targeting the PI3K pathway which are in various stages of clinical development. In many respects the PI3K pathway holds a special place in PPIns metabolism as flux through the pathway is generally very low but is strongly stimulated by oncogenic pathways. While this imparts a therapeutic potential, the involvement of the PI3K pathway in normal cellular processes ultimately leads to potential for on target toxicities. For example the PI3K pathway is intimately involved in insulin signalling and on target issues with glucose homeostasis are often seen in therapeutic treatments (89). As signalling through one PPIns can impact on multiple downstream events, there are several points within a particular PPIns pathway that could be the focus for intervention, all of which likely would yield different outcomes (**Figure 4B**). We illustrate this using a specific nuclear PPIns modulator which changes the levels of its PPIns product to impact on epigenetic signalling proteins PHD1-PHD5. Each of these then can impact on the transcription of at least two genes. The system behaves similarly to what was observed with nuclear PIP4K2B, PtdIns5P and the downstream PHD finger containing proteins ING2 (57, 58, 86) and TAF3 (59). We envisage three points for therapeutic intervention. Inhibition of the PPIns modulating enzyme attenuates signalling through all the downstream PHD finger proteins effecting transcriptional output of all genes A-L. Inhibition instead at the level of the PHD finger protein attenuates only output of gene A and B. Finally, targeting the interaction site specifically between a PHD finger and the PPIns has the potential to attenuate selective transcriptional output. In this instance two different PHD finger/PPIns interactions are targeted to selectively block the transcriptional output of only gene A and F. Similar selective outputs were observed using mutants of ING2 and TAF3 that are unable to interact with PPIns but still interact with H3k4me3 (59, 86).

Exploiting PPIns signalling complexity, however, requires deep level understanding of how these interventions impact output, the gathering of which has become much more feasible with the advent of CRISPR gene editing tools. Combining this knowledge with bioinformatic network analysis using tools such as Mogrify and knowledge of patient specific transcriptional landscapes might allow the complexity within PPIns signalling pathways to be exploited by intervening at various levels to convert an AML cell to a differentiated and non-proliferative cell state.

## Author Contributions

RF and ND contributed to the conception of the work. All authors contributed to the article and approved the submitted version.

## Conflict of Interest

The authors declare that the research was conducted in the absence of any commercial or financial relationships that could be construed as a potential conflict of interest.
